# Sexual transmission of Hepatitis C Virus infection in a heterosexual population: A systematic review

**DOI:** 10.12688/hrbopenres.12791.1

**Published:** 2018-03-08

**Authors:** Francesca Wuytack, Vittoria Lutje, Janus Christian Jakobsen, Karl Heinz Weiss, Paula Flanagan, Georgina Gethin, Louise Murphy, Siobhan Smyth, Declan Devane, Valerie Smith

**Affiliations:** 1 School of Nursing & Midwifery, Trinity College Dublin, Dublin, D02 T283, Ireland; 2 Cochrane Infectious Diseases Group, Department of Clinical Sciences, Liverpool School of Tropical Medicine, Liverpool, L3 5QA, UK; 3 Rigshospitalet Copenhagen, Copenhagen Trial Unit, Copenhagen O, DK-2100, Denmark; 4 Internal Medicine IV, Head of Section of Transplant Hepatology, Liver Cancer Center Heidelberg, Heidelberg, Germany; 5 Health Services Executive Health Protection Surveillance Centre, Dublin, D01 A4A3, Ireland; 6 School of Nursing & Midwifery, National University of Ireland Galway, Galway, Ireland

**Keywords:** hepatitis C virus, viral hepatitis, public health policy, risk factors, sexual transmission, systematic review

## Abstract

**Background:** Hepatitis C virus (HCV) infection is an important cause of liver disease worldwide. Identification of risk factors can guide screening and prevention. Sexual transmission in monogamous heterosexual relationships is rare but it is uncertain which sexual behaviours are linked to HCV transmission. This review aimed to determine risk factors for sexual HCV transmission in heterosexuals in low HCV prevalence countries (PROSPERO registration CRD42016051099).

**Methods:** We searched Medline, Embase, Science Citation Index-Expanded, Social Sciences Citation index, Conference proceedings (Web of Science), CINAHL, Scopus, LILACS, PubMed, and grey literature (04/11/2016).

We included studies published in/after the year 2000 that examined sexual risk factors for HCV infection, other than interspousal transmission, in heterosexual adults (≥18 years). We excluded prisoners, people who inject drugs (PWIDs), people co-infected with HIV or from high prevalence countries. Two reviewers completed study selection, data extraction, risk of bias and quality of evidence assessment (GRADE) independently. Meta-analysis could not be conducted.

**Results:** Eight studies were included, examining seven factors (multiple sex partners, receiving/providing sex commercially, PWID partner, and unprotected vaginal, oral, anal sex). None were significant, except the evidence for the factor having a PWID partner was conflicting.

**Conclusions:** We are uncertain about the results due to the very low quality of evidence (GRADE). A more liberal approach to review inclusion criteria might be useful in further identifying factors associated with an increased risk of sexual transmission of HCV infection in a heterosexual population. However, caution should be applied to avoid the impact of confounders on the findings.

## Introduction

Hepatitis C virus (HCV) infection was first identified in 1989 ^1^. Chronic HCV infection is an important cause of chronic liver disease and liver related death worldwide ^2^ with an estimated 130 to 150 million persons having chronic HCV infection globally ^3^. The prevalence of HCV infection varies across countries and areas ^4^. Of those infected with HCV, many are asymptomatic with approximately 20% to 30% developing acute symptoms ^5, 6^ and 55% to 85% developing chronic infection ^7^. Of those who develop chronic infection, 15% to 30% will develop liver cirrhosis within 20 years ^3^. Annually, approximately 3% to 4% of patients with liver cirrhosis develop hepatocellular carcinoma ^8^.

Identification of risk factors associated with HCV transmission is essential in guiding screening and prevention strategies to improve health outcomes and maximise cost-effectiveness. There is currently no effective vaccination for HCV, putting even more emphasis on infection prevention ^9^. Since the introduction of routine screening of blood in the early 1990s, transfusion-related HCV infection is rare. Literature on risk factors for HCV infection indicate that Injecting Drug Use (IDU) is now the main mode of transmission ^10^. Other reported risk factors include occupational exposure, tattooing or having blood transfusions, vertical transmission and sex with an infected partner ^10^.

The role of sexual transmission in HCV transmission is not fully understood and an increasing number of studies examine this question. Some studies found HCV RNA in semen ^11, 12^, but other studies have contradicted these findings ^13, 14^. Tohme and Holmberg ^15^ conducted a systematic review concerning the risk of HCV sexual transmission. They found that having multiple sexual partners might increase risk of HCV infection, although this finding may be confounded by IDU. Moreover, HIV co-infected individuals and Men who have Sex with Men (MSM) were clearly more at risk. Sexual transmission in people in monogamous heterosexual relationships on the other hand is rare; however, it is uncertain what specific sexual behaviours in heterosexuals do increase the risk of HCV transmission. Since their review was published in 2010, additional studies have emerged and an updated review was required to inform guidance on screening for HCV infection. We also aimed to increase generalisability to the context of low HCV prevalence countries because in high endemic countries there may be other risk factors in the population that make it difficult to identify sexual transmission at the source. Moreover, we focussed on heterosexuals but excluded high risk populations such as people who inject drugs (PWID), prisoners and HIV co-infected people, to address the question of when HCV screening is warranted in a more general population. Subsequently, the aim of this systematic review was to determine what factors, if any, are associated with an increased risk of sexual transmission of HCV infection in a heterosexual population in low HCV prevalence countries. This review was registered in PROSPERO ( CRD42016051099).

## Methods

### Search strategy

A comprehensive search of both electronic databases and grey literature was conducted by VL. We searched the following databases up to 4 ^th^ November 2016, attempting to identify all relevant studies: Medline (OVID), EMBASE (OVID), Science Citation Index-Expanded, Social Sciences Citation index, Conference proceedings (Web of Science), Cinahl (EBSCOHost), Scopus and LILACS (Bireme). We used a combination of controlled vocabulary terms and free-text terms including: Hepatitis C, Hepacivirus, Incidence, Prevalence, Risk-Taking, Risk Factors, Sexually Transmitted Diseases, transmission, Exposure, Sexual Behavior, Sexual Partners. We adapted the queries to each database. We did not limit our searches by time or language. We searched for additional studies by reviewing the reference lists of all included studies, and by using the “Similar articles” function in Medline. For grey literature, we looked at the following websites: WHO ( World Health Organization); CDC ( Centre for Disease Control and Prevention); ECDC ( European Centre for Disease Control and Prevention); BASHH ( British Association for Sexual Health & HIV); IUSTI ( International Union against Sexually Transmitted Infections); AASLD ( American Association for the Study of Liver Diseases); EASL ( European Association for the Study of the Liver); Society for the Study of Sexually Transmitted Diseases in Ireland ( SSSTDI); American Sexually Transmitted Diseases Association ( ASTDA). 

**Figure 1.**
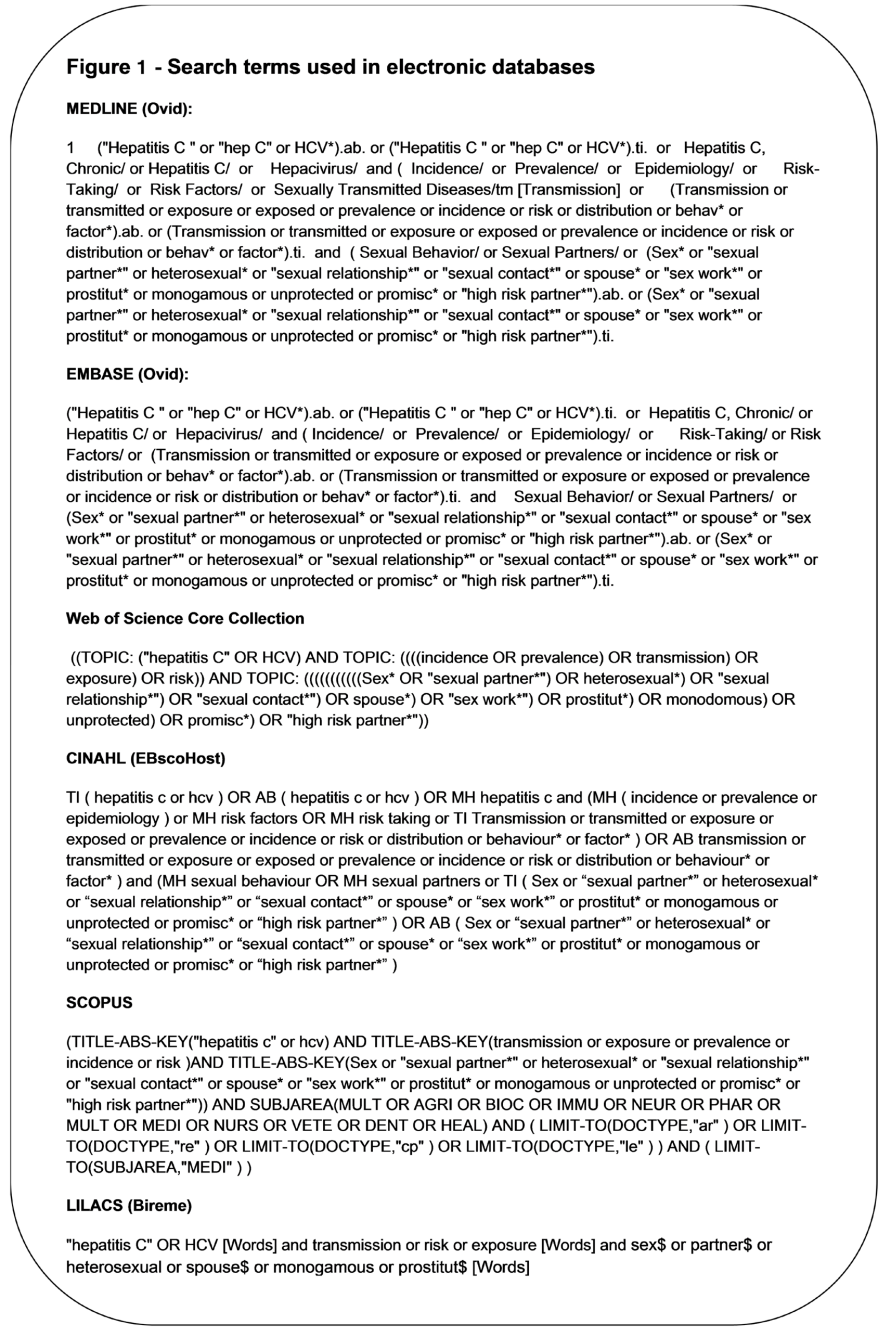
Search terms used in electronic databases.

More details on the search terms can be found in Figure 1.

### Selection criteria

The selection criteria were set in Population, Exposure, Outcome, Study design (PEOS) format. The population of interest included heterosexual adults (≥18 years), excluding those with HIV co-infection, PWIDs, homo- or bisexuals, or prisoners, because these populations are at high risk of HCV infection. In addition, this review excluded studies conducted in high HCV prevalence countries, because the aim of this review was to provide guidance for assessing the need of screening specific populations in the setting of low HCV prevalence countries ^16^. The list of high HCV prevalence countries was obtained from the Health Protection Surveillance Centre (Ireland), Infectious disease assessment for migrants ^17^, but the HCV prevalence of Nigeria was changed to high (>3%) following the publication of the epidemiological report on hepatitis C and B by the European Centre for Disease Prevention and Control in August 2016 ^16^.

The exposure was any sexual behaviour factor including (but not limited to) having multiple sex partners, overlapping (more than one sexual relationship at the same time), changing sexual partners frequently, unprotected sex outside of monogamous relationship (sex acts without the use of a condom), exchange of sex for drugs (but not PWID) or money, being a commercial sex worker, sex with commercial sex workers, sex under the influence of drugs or alcohol, anal sex, having another Sexual Transmitted Infection (STI) (excluding HIV), having a high risk partner (defined as any of the above). The exposure could be self-reported or based on an objective measure e.g. the number of occasions condoms used/not used. The outcome HCV infection had to be determined by antibody/antigen or PCR RNA test, excluding self-reported HCV status.

We included cohort studies, case-control studies and cross-sectional studies, but excluded case studies, case series and reviews. We only included studies published in or after the year 2000 because of the variability in the quality of HCV serological testing in earlier studies.

### Study selection

Records identified in the search were screened independently by title/abstract and then by full-text by at least two reviewers (FW, VS, PF, GG, LM, SS). Conflicts were resolved by FW, VS and PF through discussion, and if necessary by involving another reviewer (DD).

### Data extraction and risk of bias assessment

Risk of bias (ROB) in the included studies was assessed by two independent reviewers (FW, JCJ) using the Quality In Prognosis Studies (QUIPS) tool ^18^. The ROB criteria for each QUIPS domain specific to this review, including appropriate methods for HCV infection measurement and important confounders should have been adjusted for, were documents *a priori* and agreed by all authors. Conflicts were resolved through discussion. A data extraction form was developed and reviewed by all authors. Data extraction was conducted by two reviewers independently (VS, GG, LM and FW). Any conflicts were resolved by a third reviewer (FW).

### Data analysis

When only raw data (proportions) were available, we calculated the unadjusted Odds Ratios (OR) and 95% Confidence Intervals (CI) using the natural log scale ^19^. We planned to conduct meta-analyses in Revman ^20^ and to assess statistical heterogeneity (I ^2^ ≥ 50%, T ^2^ > 0, or the p-value > 0.10 for the Chi square test) ^21^. However, it was not appropriate to carry out meta-analysis due to clinical and methodological heterogeneity, and findings are summarised narratively and presented in evidence tables.

The quality of evidence was assessed by two independent reviewers (VS and FW) using the Grading of Recommendations Assessment, Development and Evaluation (GRADE) approach for prognostic factor research ^22^. This review was reported according to the Meta-analysis of observational studies in epidemiology (MOOSE) guidelines (completed checklist in Supplementary File 1) ^23^.

## Results

A total of 10460 records were identified through the database searches. We did not identify any records through the grey literature searches. Two further duplicates were detected and 10458 records were screened by title and abstract, of which 274 were selected as potentially eligible and assessed by full-text. A total of eight studies were finally included.

**Figure 2.**
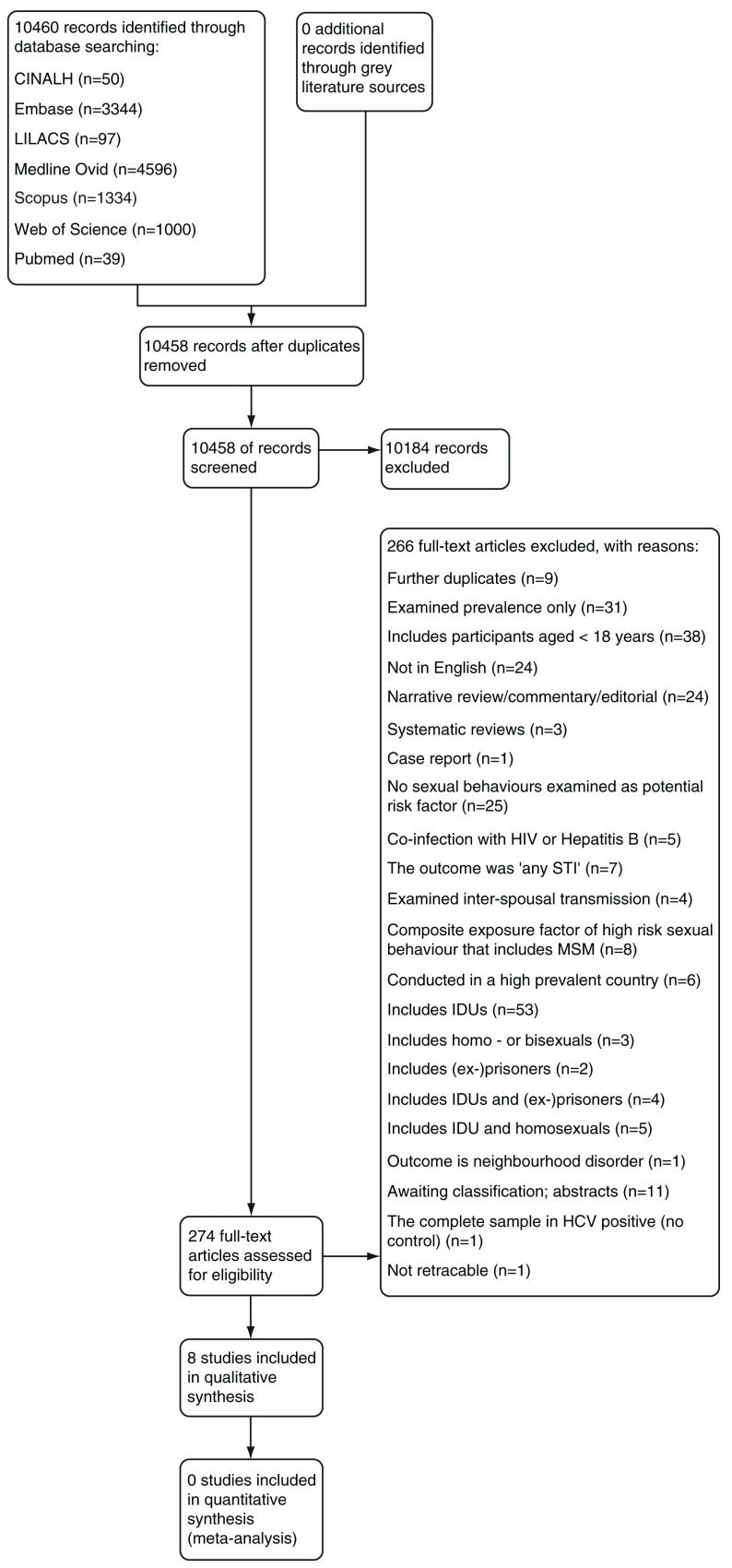
Search and selection flow diagram.

We contacted the authors to obtain more information to assess eligibility for an additional 11 records in abstract format and these are awaiting classification. Full details of the search results and selection process are presented in Figure 2.

### Characteristics of included studies

The 8 included studies were published between the year 2000 and 2015, in Mexico (n=2), USA (n=2), Vietnam (n=1), Scotland (n=1), Gambia (n=1), and Brazil (n=1). A total of 14036 participants were included in the eight studies and seven potential sexual risk factors were assessed across the studies. These were multiple sex partners, receiving or providing sex commercially, having a PWID (People who inject drugs) partner, and unprotected vaginal, oral or anal sex.

Only three factors were examined in more than one study. The majority of studies included sample populations from specific groups: one study examined risk factors in a sample of blood donors ^24^, one involved pregnant women attending antenatal services ^25^, one in homeless people ^26^, one in non-PWIDs ^27^, one in nurses ^28^, and one in a sample of different risk groups ^29^. Full details of the characteristics of the included studies are presented in Table 1.

**Table 1.** Characteristics of included studies.

Study (Design)	Setting (Country)	Participant inclusion/exclusion criteria	Risk factors (measurement method)	Outcome measurement
Dunford *et al*. (2012) ^31^ (Cross-sectional)	Different geographic regions of Vietnam	8 different population groups including people who inject drugs (PWIDs), commercial sex workers (CSWs), blood donors, military recruits, pregnant women, dialysis patients, elective surgery patients and recipients of multiple blood transfusions. ^a^	Commercial sex worker (unclear)	Enzyme immunoassay (EIA) for HCV using the Monolisa Ag/Ab HCV Ultra (Bio-Rad Laboratories, CA, USA).
Goldberg *et al*. (2001) ^25^ (Retrospective)	Ninewells Hospital Dundee; antenatal clinic (Scotland)	All women who were seen at the antenatal clinic and all women who were admitted for termination of pregnancy to the gynaecology wards.	Non-PWID but partner is PWID (Linked dataset information)	Initial screening by Ortho Diagnostics hepatitis C virus 3.0 ELISA Assay. If HCV antibodies identified, retested using Monolisa hepatitis C virus confirmatory testing; only serum samples which were reactive on both tests were considered to be HCV antibody positive.
Mboto *et al*. (2005) ^24^ (Cross-sectional)	Royal Victorial Hospital, Banjul (Gambia)	Asymptomatic first-time blood donors.	Polygamous marriage	ELISA assay system for analysis of blood samples.
Melo *et al*. (2015) ^30^ (Cross- sectional)	Rural urban area - population 2,640 inhabitants (Brazil)	> 18 years and providing informed consent for interview and blood sampling	≥6 sexual partners over a lifetime, ≥2 sexual partners over a lifetime (Structured interview questionnaire)	Enzyme immunoassay tests to detect the markers anti-HCV. Qualitative detection of hepatitis C virus- ribonucleic acid (HCV RNA) [Amplicor version 2.0 (Roche)] was performed for the anti-HCV-positive serum or potentially positive serum.
Mendez-Sanches *et al*. (2005) ^29^ (Cross-sectional)	University Hospital Check-up unit, Mexico City (Mexico)	Inclusion: people with one of the following risk factors: blood transfusion before 1992; surgeries before 1992; IV drug use as unique risk factors. People with two or more the following risk factors: tattoos, contact with known HCV- infected people; previous manicures or pedicures with a non-personal instrument; dental surgery; piercing; acupuncture; more than three sexual partners ^b^	More than three sexual partners (Patients were interviewed and completed a questionnaire)	Screened for HCV RNA by qualitative polymerase chain reaction (PCR) using the Cobas Amplicor HCV Test Version 2.0 (Roche Laboratories Ltd., USA). In HCV RNA-positive patients, genotyping was performed using the HCV RNA Genotype Duplitype Assay (Quest Diagnostics, USA).
Mendez-Sanches *et al*. (2006) ^28^ (Cross-sectional)	Tertiary-care hospital, Mexico City (Mexico)	All nursing personnel.	More than four Sexual partners (Self-reported questionnaire)	Axsyum HCV system version 3. In positive cases qualitative and quantitative viral load and genotype were assessed through PCR for RNA- HCV.
Neaigus *et al*. (2007) ^27^ (Prospective longitudinal)	New York City (USA)	>18 years, had used non-injected heroin during the preceding 30 days, and had either never injected drugs or had not done so during the preceding 6 months. Eligibility tests of urine, hair, body sites, structured screening questionnaire and ethnographic methods were used.	Unprotected vaginal sex, Unprotected anal sex, Unprotected oral sex, Commercial sex received, Commercial sex provided, Multiple sex partners in last 30 months, Sex with PWID (structured interview questionnaire)	HCV antibody tested by HCV EIA 2.0
Nyamathi *et al*. (2002) ^26^ (Cross-sectional but sample derived from a quasi-experimental study)	36 homeless shelters or sober- living shelters or from street outreach in Los Angeles (USA)	18–65 years; homeless; having an intimate partner or friend termed an impoverished adult.	Multiple sex partners in last 6 months (No versus yes) (structured questionnaire)	Ortho HCV ELISA Test kit System Version 3.0

^a^Includes some PWIDs but prevalence of non-PWIDs reported.

^b^Even though PWIDs were not an exclusion criteria, no participants were PWIDs.

### Characteristics of excluded studies

A total of 266 studies were excluded at full-text selection. Reasons for exclusion included participants under the age of 18 in the sample (n=38), study examined the prevalence of HCV but did not assess risk factors (n=31), and study publication not available in English (n=24), which may have introduced language bias. Many studies examined risk factors other than sexual factors of interest (n=25). Twenty-eight were excluded because of their study design; 24 were narrative reviews, three were systematic reviews, and one study was a case report. Sixty-seven studies included PWIDs, homosexuals and/or (ex-) prisoners in their sample and did not report findings separately for these groups. 

### Risk of bias of included studies.

Details of the judgment of ROB of each domain for each study are provided in Table 2. Study participation was judged as moderate (n=2) or high (n=6) ROB for all studies. Participants of included studies were specific groups that might have influenced the findings, such as healthy blood donor (n=1), nurses (n=1), pregnant women (n=1), homeless people (n=1). One study had a low recruitment rate ^30^, but other studies did not describe their recruitment rate.

**Table 2.** Risk of bias of included studies.

Record	Study participation ^a^	Study attrition ^a^	Factor measurement ^a^	Outcome measurement ^a^	Study confounding ^a^	Statistical analysis and reporting ^a^
Dunford *et al.* (2012) ^31^	+	-	+/-	-	+	+
	Recruitment not described, no baseline characteristics for all participants.	Cross- sectional study	No clear method of measurement provided.	Enzyme immunoassay (EIA) for HCV using the Monolisa Ag/ Ab HCV Ultra (Bio-Rad Laboratories, CA, USA).	No adjustment for confounders	No multivariable analysis.
Goldberg *et al.* (2001) ^25^	+/-	-	-	-	+	+
	Selective sample of onlly pregnant women.	Cross- sectional study	Structured questionnaire	Ortho Diagnostics hepatitis C virus 3.0 ELISA Assay (Chiron Corporation, Emeryville, California)	No adjustment for confounders	No multivariable analysis.
Mboto *et al.* (2005) ^24^	+	-	+/-	-	+	+
	Only healthy blood donors included of which only 2 women.	Cross- sectional study	Interviews not clearly described.	ELISA kit	No adjustment for confounders	No multivariable analysis.
Melo *et al.* (2015) ^30^	+/-	-	-	-	-	-
	Only 51.9% participation rate	Cross- sectional study	Structured standardised questionnaire	virusribonucleic acid (HCV RNA) [Amplicor version 2.0 (Roche)] was performed for the anti-HCV-positive serum or potentially positive serum. Virusribonucleic acid (HCV RNA) [Amplicor version 2.0 (Roche)] was performed for the anti-HCV-positive serum or potentially positive serum.	Adjustment for confounders	Multivariable analysis. Clearly reported.
Mendez-Sanches *et al.* (2005) ^29^	+	-	-	-	+	+
	Only includes participants who report certain risk factors	Cross- sectional study	structured questionnaire	Qualitative polymerase chain reaction (PCR) using the Cobas Amplicor HCV Test Version 2.0 (Roche Laboratories Ltd., USA). HCV RNA-positive serum was also screened by quantitative PCR using the Cobas Amplicor HCV Test Version 2.0(Roche Laboratories Ltd) with a dynamic range lower limit of 50 IU/mL. In HCV RNA-positive patients, genotyping was performed using the HCV RNA Genotype Duplitype Assay (Quest Diagnostics, USA), a DNA sequencing technology to subtype two regions of the HCV genome:the CORE gene and the NS5B region.	No adjustment for confounders	No multivariable analysis.
Mendez-Sanches *et al.* (2006) ^28^	+	-	-	-	-	+
	Only nurses included	Cross- sectional study	Structured questionnaire	Axsyum HCV system version 3	Adjustment for confounders	Multivariable analysis but not clearly reported.
Neaigus *et al.* (2007) ^27^	+	+	-	-	-	-
	Only (non-intravenous) drug users	Only 62.2% retention rate.	Structured interview	HCV EIA 2.0 (Abbott)	Adjusted for important confounders	Multivariable analysis and clearly reported.
Nyamathi *et al.* (2002) ^12^	+	-	-	-	+/-	+/-
	Only homeless people included.	Cross- sectional study	structured questionnaire	ELISA kit	Adjusted for gender, age, age started living on their own, daily alcohol use but not adjusted for tattoo/ body piercing	Clearly presented and multivariable analysis but key confounders not included.

^a^Risk of bias for each domain was judges as high (+), moderate (+/-) or low (-).

Seven of the 8 included studies were cross-sectional studies, hence there was low attrition bias. Only Neaigus *et al*. ^27^ followed up patients and had a low retention rate of only 62.2%, leading to high risk of attrition bias. Six of the 8 studies were judged as low ROB for the domain risk factor measurement as the risk factor was measured using a structure questionnaire. Two studies were of moderate ROB because they did not provide a clear description of risk factor measurement.

All studies appropriately assessed the outcome HCV infection, most commonly using the ELISA kit, and were thus judged as low ROB. Four studies did not adjust for confounders and were therefore judged as high ROB for this domain. Nyamathi *et al*. ^26^ adjusted for confounders but did not include some important confounders such as a history of tattooing and was judged as moderate ROB. The remaining three studies were judged low ROB. Four studies did not conduct multivariable analysis to adjust for confounders and hence were rated as high ROB for statistical analysis. Nyamathi *et al*. ^26^ adjusted for some confounders (gender, age, age started living on their own, daily alcohol use) but not all important ones (e.g. tattooing/body piercing) were included in the model; hence this study was rated as moderate ROB.

### Risk factors

A total of seven potential risk factors were examined in the eight included studies. Evidence for all factors was of very low quality and full details of the GRADE profiles by factor are provided in Table 3.

**Table 3.** GRADE profile of risk factors examined in included studies.

Potential risk factor identified	No. of participants	Reference(s) & phase of investigation	No. of studies	Univariate			Multivariate			Dominant Phase**	GRADE factors ^k^							
				+	0	-	+	0	-		Study limitations	Inconsistency	Indirectness	Imprecision	Publication bias	Moderate/ large effect size	Dose effect	Overall quality ^h^
Multiple sex partners	2884	Neaigus *et al*. (2007) (Phase 1) ^i^ Nyamathi *et al*. (2002) (Phase 1) ^j^ Melo *et al*. (2015) (Phase 1) ^i^ Mendez-Sanchez *et al*. (2005) (Phase 1) ^i^ Mendez-Sanchez *et al*. (2006) (Phase 1) ^i^ Mboto *et al*. (2005) (Phase 1) ^i^	6	0	6	0	0	1	0	1	x ^a^	v	v	x ^b^	v	v	v	+
Commercial sex work	7931	Dunford *et al*. (2012) (Phase 1) ^i^ Neaigus *et al*. (2007) (Phase 1) ^i^	2	0	2 ^g^	0	0	0	0	1	xx ^c^	x ^d^	v	x ^b^	x ^d^	v	v	+
History of sex with CSW	277	Neaigus *et al*. (2007) (Phase 1) ^i^	1	0	1	0	0	0	0	1	xx ^c^	v	v	x ^b^	x ^d^	v	v	+
Partner who is an IDU	3775	Neaigus *et al*. (2007) (Phase 1) ^i^ Goldberg *et al*. (2001) (Phase 1) ^i^	2	1	1	0	0	0	0	1	x ^f^	x ^e^	v	x ^b^	x ^d^	v	v	+
Unprotected vaginal sex	277	Neaigus *et al*. (2007) (Phase 1) ^i^	1	0	1	0	0	0	0	1	x ^f^	x ^d^	v	v	x ^d^	v	v	+
Unprotected anal sex	277	Neaigus *et al*. (2007) (Phase 1) ^i^	1	0	1	0	0	0	0	1	x ^f^	x ^d^	v	v	x ^d^	v	v	+
Unprotected oral sex	277	Neaigus *et al*. (2007) (Phase 1) ^i^	1	0	1	0	0	0	0	1	x ^f^	x ^d^	v	v	x ^d^	v	v	+

^a^ Downgraded by one level because seven of the eight studies had at least one domain high ROB or two domains moderate ROB; ^b^ Downgraded by one level because some studies have wide confidence intervals and no power calculations provided; ^c^ Downgraded two levels since the study has more than 1 domain high ROB or two moderate; ^d^ Downgraded by one level because only one or two study(ies) has(ve) provided an effect estimate; ^e^ Downgraded by one level because the confidence intervals do not overlap; ^f^ Downgraded by one level because of a study has more than 1 domain high ROB or two moderate; ^g^ For one of the two studies the effect could not be estimated and was subsequently classified as no effect; ^h^ GRADE levels of evidence: + very low, ++ low, +++ moderate, ++++ high quality; ^i^ Phase 1 of investigation (Hayden 2008), conducted only univariate analysis for the factor of interest; ^j^ Phase 1 of investigation (Hayden 2008), multivariable analysis but no specific hypotheses tested; ^k^ Explanation of symbols: ‘v’ not downgraded/upgraded, ‘x’ downgraded/upgraded by one level, ‘xx’ downgraded/upgraded by two levels.

Six studies examined having had multiple sex partners as a potential risk factor for HCV infection ( Table 4). Different cut-offs and analysis methods did not allow us to pool data in meta-analysis. Only one study adjusted for confounders and did not find having more than three sex partners in the last six months to be a significant risk factor ^26^. Similarly, the other five studies did not find a positive association. However, evidence should be interpreted with caution since the quality of evidence (GRADE) is very low.

**Table 4.** Multiple sex partners as a risk factor for Hepatitis C infection.

Factor	Study	No of participants in analysis	Risk estimate (unadjusted)	Adjusted risk estimate	Confounders adjusted for
Multiple sex partners in the last 30 months	Neaigus *et al*. (2007) ^27^	277	OR ^a^ 0.9 (0.4–2.1), p=0.8	/ ^b^	N/A
> 3 sex partners in last 6 months (No versus yes)	Nyamathi *et al*. (2002) ^26^	743 non- injection drug user samples	OR 0.2 (0.03 to 1.5)	AOR 0.14 (0.02 to 1.06)	Gender, age, ethnicity, age started living alone, recent daily alcohol use.
≥ 2 sexual partners over a lifetime	Melo *et al*. (2015) ^30^	1001	One (0.1%) case of confirmed HCV infection. This man denied blood transfusion, He reported no more than 5 sexual partners over a lifetime or 2 partners in the last 6 months.	/ ^b^	N/A
> 3 sexual partners	Mendez- Sanchez, *et al*. (2005) ^29^	300	OR ^a^ 1.6 (0.3–8.1), p=0.6	/ ^b^	N/A
> 4 sexual partners	Mendez- Sanchez, *et al*. (2006) ^28^	376	OR ^a^ 1.5 (0.08–29.7), p=0.8	Not included in multivariable analysis due to insignificant in univariate analysis.	N/A
≥ 6 sexual partners over a lifetime	Melo *et al*. (2015) ^30^	1001	One (0.1%) case of confirmed HCV infection. This man denied blood transfusion, He reported no more than 5 sexual partners over a lifetime or 2 partners in the last 6 months.	/ ^b^	N/A
Polygamous marriage (vs monogamous marriage)	Mboto *et al*. (2005) ^24^	187	OR 2.6 (0.24–27.8)	/ ^b^	N/A

^a^Calculated from raw data (95% CI calculated using natural logarithm method ^19^.

^b^Adjusted risk estimate not available.

Evidence of being a commercial sex worker as a risk factor for HCV infection was limited (measured in 2 studies only ^27, 31^) and of very low quality (GRADE). Dunford *et al*. ^31^ found that 8.7% of commercial sex workers were HCV positive, and of those 40.2% (n=87) were non-PWIDs, but did not report data on the comparison group (non-PWID CSWs negative for HCV). Neaigus *et al*. ^27^ found a positive effect but it was not statistically significant (unadjusted OR 2.0 (0.6-6.7); p=0.3; n=277).

A history of having sex with a commercial sex worker was not associated with HCV infection (unadjusted OR 1.9 (0.5-8.0), p=0.4; Male: HR 4.1 (0.91–18.0); Female: not reported; n=277; one study), but the quality of evidence (GRADE) was very low due to only one study examining this factor with significant risk of bias ^27^.

There was conflicting evidence regarding the role of having a partner who is a PWID as a risk factor for HCV infection and quality of evidence was very low due to significant ROB and a limited number of studies examining this factor. Goldberg *et al*. ^25^ found that having a PWID partner was a significant risk factor (unadjusted OR 56.6 (18.5 -173.60), p<0.0001; n=3498), but Neaigus *et al*. ^27^ found no association (unadjusted OR 1.2 (0.3 –5.2); n=277).

In one study (n=277), unprotected vaginal sex (unadjusted OR 1.5 (0.8–2.7), p=0.2; Males: Hazard Ratio (HR) 0.75 (0.25–2.3); Females: HR 0.49 (0.11–2.3)), unprotected anal sex (unadjusted OR 0.8 (0.2–3.1), p=0.8; Females: HR 1.7 (0.22–12.8); Male: not reported), and unprotected oral sex (unadjusted OR 0.7 (0.4–1.3), 0.2; Females: HR 0.93 (0.21–12.8); Male: not reported) were not associated with HCV infection (very low quality evidence) ^27^.

## Discussion

Seven potential sexual risk factors for HCV transmission in a heterosexual population were examined in eight studies, including multiple sex partners, receiving or providing sex commercially, having a PWID partner, and unprotected vaginal, oral or anal sex. None of these factors were statistically significant risk factors in the included studies; however, we are uncertain about these results due to the very low quality of evidence (GRADE). Moreover, these results might have been affected by a potential lack of statistical power and none of the studies provided sample/power calculations. Goldberg *et al*. ^25^ did find that having a sex partner who is a PWID was associated with an increased risk of HCV infection resulting in conflicting evidence because the other study examining this factor did not find a significant association ^27^. Goldberg *et al*. ^25^ was conducted in Scotland, which might provide more geographically relevant information as this is a similar context to Ireland and other European countries. However, being a partner of a PWID could also expose people to non-sexual HCV transmission and may have confounded this finding.

Evidence for all factors examined in the included studies was of very low quality, mainly due to a lack of replication and high ROB resulting from a lack of adjusting for confounders and selective samples. Incomplete or non-standardised measurement of sex practices in some of the included studies could also have impacted on the findings of this review. Moreover, only seven factors were examined in the included studies and other factors such as use of condom, sex during menses, rough sex, presence of other STIs etc., were not measured and assessed. Even though we excluded studies that examined PWIDs, subjects may not disclose being a PWID, particularly since data on factors was generally obtained through a self-reported questionnaire.

The strengths of this review lie in its comprehensive search, its double independent study selection, ROB assessment, data extraction and GRADE quality assessment.

This review adhered to the *a priori* selection criteria set and excluded any study that included PWIDs, prisoners and/or homo- or bisexuals in their sample in the absence of subgroup analyses for these groups. This approach limited the number of included studies. A more liberal approach to review study inclusion criteria (i.e. including the 67 studies that partly included these groups) might be useful in further addressing the objective of this review. However, caution should be had when doing so to avoid the impact of confounders on the findings and we would recommend conducting subgroup analyses in such case.

## Data availability

All data underlying the results are available as part of the article and no additional source data are required.

## Supplementary material

Supplementary File 1: MOOSE checklist.
