# Supplementary material for: Sexual transmission of Hepatitis C Virus infection in a heterosexual population: A systematic review
Source: HRB Open Res. 2018 Mar 8;1:10. [Version 1] doi: 10.12688/hrbopenres.12791.1 (PMC6973529; doi:10.12688/hrbopenres.12791.1)
Supplement: Supplementary file 1 [file hrbopenres-1-13851-s0000.tgz › 21344a32-66ca-42d7-bdca-d8d0c1ea1c80.doc]

**MOOSE Checklist**

| **Item No** | **Recommendation** | **Reported on Page No** |
| --- | --- | --- |
| Reporting of background should include | | |
| 1 | Problem definition | 5-6 |
| 2 | Hypothesis statement | 5-6 |
| 3 | Description of study outcome(s) | 7-8 |
| 4 | Type of exposure or intervention used | 7-8 |
| 5 | Type of study designs used | 7-8 |
| 6 | Study population | 7-8 |
| Reporting of search strategy should include | | |
| 7 | Qualifications of searchers (eg, librarians and investigators) | 1 |
| 8 | Search strategy, including time period included in the synthesis and key words | 6-7+fig1 |
| 9 | Effort to include all available studies, including contact with authors | 6-7+fig 1 |
| 10 | Databases and registries searched | 6-7+fig 1 |
| 11 | Search software used, name and version, including special features used (eg, explosion) | 6-7+fig 1 |
| 12 | Use of hand searching (eg, reference lists of obtained articles) | 6-7 |
| 13 | List of citations located and those excluded, including justification | Figure 2 |
| 14 | Method of addressing articles published in languages other than English | 6-7 |
| 15 | Method of handling abstracts and unpublished studies | 6-7 |
| 16 | Description of any contact with authors | 6-7 |
| Reporting of methods should include | | |
| 17 | Description of relevance or appropriateness of studies assembled for assessing the hypothesis to be tested | 10 (+table 2) |
| 18 | Rationale for the selection and coding of data (eg, sound clinical principles or convenience) | 9 |
| 19 | Documentation of how data were classified and coded (eg, multiple raters, blinding and interrater reliability) | 9 |
| 20 | Assessment of confounding (eg, comparability of cases and controls in studies where appropriate) | 9 |
| 21 | Assessment of study quality, including blinding of quality assessors, stratification or regression on possible predictors of study results | 9 |
| 22 | Assessment of heterogeneity | 9 |
| 23 | Description of statistical methods (eg, complete description of fixed or random effects models, justification of whether the chosen models account for predictors of study results, dose-response models, or cumulative meta-analysis) in sufficient detail to be replicated | 9 |
| 24 | Provision of appropriate tables and graphics | Figure 1+2, tables 1-4 |
| Reporting of results should include | | |
| 25 | Graphic summarizing individual study estimates and overall estimate | Table 4 |
| 26 | Table giving descriptive information for each study included | Table 1 |
| 27 | Results of sensitivity testing (eg, subgroup analysis) | Unable to perform |
| 28 | Indication of statistical uncertainty of findings | 10-11+table 4 |

| **Item No** | **Recommendation** | **Reported on Page No** |
| --- | --- | --- |
| Reporting of discussion should include | | |
| 29 | Quantitative assessment of bias (eg, publication bias) | Unable to perform |
| 30 | Justification for exclusion (eg, exclusion of non-English language citations) | Figure 2 |
| 31 | Assessment of quality of included studies | Table 3 |
| Reporting of conclusions should include | | |
| 32 | Consideration of alternative explanations for observed results | 13-15 |
| 33 | Generalization of the conclusions (ie, appropriate for the data presented and within the domain of the literature review) | 13-15 |
| 34 | Guidelines for future research | 14-15 |
| 35 | Disclosure of funding source | 16 |

*From*: Stroup DF, Berlin JA, Morton SC, et al, for the Meta-analysis Of Observational Studies in Epidemiology (MOOSE) Group. Meta-analysis of Observational Studies in Epidemiology. A Proposal for Reporting. *JAMA*. 2000;283(15):2008-2012. doi: 10.1001/jama.283.15.2008.
