# Supplementary material for: Sexual transmission of Hepatitis C Virus infection in a heterosexual population: A systematic review
Source: HRB Open Res. 2018 Mar 8;1:10. [Version 1] doi: 10.12688/hrbopenres.12791.1 (PMC6973529; doi:10.12688/hrbopenres.12791.1)
Supplement: Peer review history

# Peer review: Reviewer response for version 1

**DOI:** 10.21956/hrbopenres.13851.r26294

**Published:** 2019-01-08

**Authors:** F. DeWolfe Miller

**Affiliations:** 1 John A. Burns School of Medicine, University of Hawaii, Honolulu, HI, USA

I have a few brief comments that may / should be addressed. Given that this can be easily done, I recommend that the manuscript be accepted for indexing.

                                          ______________________

 Re: Introduction - second paragraph: regarding transmission, an additional reference should be included for iatrogenic transmission, a significant but often overlooked route of transmission and the major driver of the HCV epidemic in Egypt.

 Re: the study is appropriately registered PROSPERO (CRD42016051099). A necessary study criterion.

 Discussion: I do not think that “of other STIs etc.,” should be included in peer reviewed scientific literature. Just reword accordingly.

 Discussion: should the authors include in the Discussion that no discordant monogamous studies were found? Or that had been previously reviewed?

 In general, this is a thorough up to date systemic review (following conventional guidelines for systemic review) of the role of sexual transmission of HCV infection. The findings, i.e. the lack of evidence for sexual transmission in low HCV prevalence communities is consistent with current literature, the lack of consistent isolation of HCVRNA from human genital track fluids, and the lack of evidence of transmission in monogamous discordant couples over time. Until concrete evidence to the contrary emerges, HCV is a blood borne pathogen. Thank you for this opportunity for service to HRB Open Research.

I confirm that I have read this submission and believe that I have an appropriate level of expertise to confirm that it is of an acceptable scientific standard.

# Peer review: Reviewer response for version 1

**DOI:** 10.21956/hrbopenres.13851.r26246

**Published:** 2018-05-29

**Authors:** Lisa Maher

**Affiliations:** 1 Kirby Institute for Infection and Immunity, UNSW Sydney, Sydney, NSW, Australia

This systematic review examined studies of sexual risk factors for HCV infection, other than interspousal transmission, in heterosexual adults.  It excluded prisoners, people who inject drugs, people co-infected with HIV and people from high prevalence countries. Two reviewers completed study selection, data extraction, risk of bias and quality of evidence assessment (GRADE) independently. Meta-analysis was not conducted and the authors recommend that future studies use expanded inclusion criteria.

 The manuscript is well written (note: the acronym PWID is plural) and organized, uses a standardised approach (MOOSE guidelines with checklist available), assesses ROB (QUIPS) and quality of evidence (GRADE).

 Results are not surprising with 8 very low quality studies (7 cross-sectional) included and high ROB as a result of lack of adjustment for confounders and selective samples. Additional limitations include incomplete or non-standardised measurement of sex practices in some studies and the failure to measure or assess potentially important factors including condom use, sex involving blood and the presence of other STIs.

 While the inclusion of groups such as people who inject drugs and gay and bisexual men in future reviews may increase the number and quality of studies, the impact of confounders is likely to be significant.

I confirm that I have read this submission and believe that I have an appropriate level of expertise to confirm that it is of an acceptable scientific standard.
